# Book Review

**DOI:** 10.4269/ajtmh.17-1003

**Published:** 2018-03

**Authors:** Philip J. Rosenthal

**Affiliations:** Department of Medicine; University of California, San Francisco; P.O. Box 0811; San Francisco, CA 94143; E-mail: philip.rosenthal@ucsf.edu

With a plethora of sources of information available on any topic, books are arguably not as important as they used to be. But we still need books to cover major topics in depth, especially topics off the beaten path, so relatively unrepresented by electronic sources. But big books can be expensive. With this background, an important event for the tropical medicine community has been the publication of the sixth edition of *Parasitic Diseases*. This release is opportune, as the book offers one-stop shopping for an updated and comprehensive overview of the broad topic of parasitology. And the release is noteworthy because, remarkably, the book is available as a PDF at no charge to anyone who asks for it. We will return to consideration of how the book is so easily available below.

The new edition of *Parasitic Diseases* has been overdue. The previous edition was published in 2005 (the first was in 1982), and there have been great advances in our understanding of parasitology since then. As noted by the authors, the book has been extensively updated, with over 1,000 new references (although perhaps too many old references were retained), and discussions of disease mechanisms, diagnostic techniques, treatments, and preventive measures are all updated. The book offers over 500 pages of text plus appendices, with extensive illustrations and profuse referencing. Chapters cover all the protozoans and helminths that are typically considered parasitic pathogens. *Parasitic Diseases* is well organized, and in deciding where to put different pathogens, the authors were willing to stick their necks out a bit. The chapter entitled “Protozoa of minor medical importance” includes a number of potentially dangerous pathogens, including *Babesia*, free amebas, and some enteric gastrointestinal pathogens; those particularly interested in these organisms might be offended by the title, and taxonomists might dislike lumping distantly related organisms. Similarly, there are chapters on nematodes, tapeworms, and trematodes of “minor medical importance.” Another interesting and well-written chapter concerns “Aberrant nematode infections”; the pathogens covered in this chapter are zoonotic, infect humans only incidentally, and cannot complete their life cycles in humans, but nonetheless, a number of them cause significant disease. Although there might be some disagreements about relative importance of different parasites, the clear organization is helpful, allowing those new to parasitology to focus on the most important pathogens. In addition to traditional parasitic protozoan and helminth pathogens, *Parasitic Diseases* covers ectoparasites, with chapters on insects and arachnids. These concern both bugs that directly cause illness and the arthropod vectors that transmit many of the most important parasitic diseases of humans. Addressing these vectors in detail is a strength of *Parasitic Diseases*. Another strength of the book is attention to the rich history of parasitology, including numerous photographs and brief bios of past giants of this field.

Even a large textbook has limitations on space, and authors must make hard choices. Readers may debate the choice of emphasis in various chapters. As an example, the chapter on malaria is comprehensive, with 32 pages covering history, biological differences among plasmodial species, cellular and molecular mechanisms of pathogenesis, clinical aspects, diagnosis, treatment, and control. The major topics are all covered, but I have some quibbles. Human malaria caused by *Plasmodium knowlesi* is now recognized to be important, but only in a circumscribed region of Asia; mention of the geographic range of this parasite would have been helpful. Cerebral malaria is not caused by the blockage of cerebral capillaries, as described, but rather by complex effects of parasites adhering in the vasculature. Clearer distinction between uncomplicated malaria and the much less common severe malaria syndromes would have been helpful. A more comprehensive discussion of artemisinin-based combination therapies, the gold standard to treat uncomplicated falciparum malaria, would have been appreciated. More attention to the two vector-based interventions most important across Africa, insecticide-impregnated bednets and indoor residual spraying of insecticides, could have been included. The discussion of vaccines is excellent in general but would have benefited from specific mention of leading approaches that may lead to approved products in the near future. One error is a statement that PfHRP2, an antigen commonly used in rapid diagnostic tests for falciparum malaria, is present in all species; in fact, it is only found in *Plasmodium falciparum*. Despite these issues, the malaria chapter does an excellent job of clearly describing the pathogen and the disease, including some challenging areas. As an example of nice communication, the authors effectively describe the confusing ancient nomenclature of malaria, which has baffled many a student, whereby fever every 2 days is referred to as tertian, and every 3 days as quartan. Other chapters similarly offer substantive, well-referenced descriptions of the major parasitic pathogens and the diseases that they cause.

The appendices in *Parasitic Diseases* detail selected diagnostic procedures, offer a diagnostic color atlas of parasitology with dozens of photographs of protozoans and helminth eggs, and display 36 life cycles (these cycles are duplicates; they are also included in their relevant chapters). Parasitologists love life cycles, and those in the book are nicely drawn. The book has an index, but it is rather small; a more detailed index would have helped *Parasitic Diseases* to compete with electronic sources, especially because users of textbooks often do not read full chapters, but rather seek out specific narrow topics with focused searches. More ambitiously, might *Parasitic Diseases* evolve into an interactive document, offering the best of a rigorous textbook plus the benefits of an online site?

*Parasitic Diseases* is not published by a large medical publisher, but rather by Parasites Without Borders (PWB). Parasites Without Borders is a nonprofit organization focused on improving education on parasitic diseases, and thereby helping underserved populations address the challenge of these diseases (www.parasiteswithoutborders.com). A key program of PWB is the dissemination of *Parasitic Diseases*. A PDF of the book is available on the PWB website at no cost. Yes, a free book! A Spanish edition is also available. A print version of the book is reasonably compact despite its breadth, with high-quality printing, illustrations, and figures. The book has been widely donated to medical schools and is sold for $70, far below the cost expected for a large medical textbook. Parasites Without Borders is also developing an online course in parasitology that will be available free of charge.

*Parasitic Diseases* is easy to acquire, but who should use it? The book seems appropriate for all those interested in in-depth exploration of pathogenic parasites. This would include undergraduates, students in medicine and related disciplines, and graduate students in parasitology or public health. The book will also serve as an excellent text for the bookshelf (or electronic shelf) of more senior parasitologists; clinicians with focus on travel, infectious diseases, or international medicine; those pursuing international public health, clinical, or medical research activities; and others seriously interested in parasitology. It is gratifying that efforts are being made to disseminate the book widely at no cost. Parasites Without Borders has provided a valuable service, and junior and senior parasitologists are encouraged to acquire the no-cost PDF or reasonably priced print version of this textbook.

